# Sequences of sickness absence, disability pension and unemployment four years before and five years after musculoskeletal diagnosis among Swedish twins

**DOI:** 10.1177/14034948241284041

**Published:** 2024-10-31

**Authors:** Annina Ropponen, Emma Pettersson, Jurgita Narusyte, Pia Svedberg

**Affiliations:** 1 Division of Insurance Medicine, Department of Clinical Neuroscience, Karolinska Institutet, Stockholm, Sweden; 2 Finnish Institute of Occupational Health, Helsinki, Finland

**Keywords:** Musculoskeletal disorders, sick leave, pension, disability, cohort, work, twins

## Abstract

**Aims:**

To investigate sustainable working life via identification of time-related sequences of sickness absence (SA), disability pension (DP) and unemployment four years before and five years after the first musculoskeletal diagnosis in a Swedish twin cohort. Other aims were to account for familial confounding and to examine the associations between sequences and sociodemographic characteristics.

**Methods:**

Among 28,474 Swedish twins, the patterns of interruptions of working life four years before the first M00-M99 diagnosis (MSD) and five years after MSD diagnosis were investigated with a sequence analysis in a seven-element state space consisting of sustainable working life, unemployment >90 days, moderate SA/DP (30–179 days), almost full year of SA/DP (180–365 days), full year of SA/DP (⩾ 365 days), death, and old-age pension.

**Results:**

The six-cluster solution had the best fit to the data. Five clusters had varying patterns of interruptions of sustainable working life (Clusters 2–6, *n* = 537–1949 with SA/DP, unemployment, but also accounting death and old-age pension) compared with the largest cluster with primarily sustainable working life (*n* = 23,316). Age, sex and familial factors affected the likelihood of belonging to the clusters with SA/DP.

**Conclusions:**

**Most Swedish twins with or without MSD diagnosis have a sustainable working life, although MSD was both prevalent and a strong risk factor for belonging to the clusters with SA/DP. Thus, early prevention of MSD and prevention of recurrent or long sickness absences due to any cause would be merited while paying special attention to women also.**

## Background

Sustainable working life, in this study, is defined as not having – or having only a few – interruptions of working life due to unemployment, sickness absence (SA) or disability pension (DP) [1], is a policy-relevant goal due to the increasing need to prolong working careers and due to the increasing aging population, which challenges the maintenance of welfare societies [2]. On the other hand, chronic diseases such as mental, musculoskeletal and cardiovascular disorders are known to cause the majority of years lost due to disability [3]. Musculoskeletal disorders, defined by the World Health Organization’s International Classification of Diseases version 10 (ICD-10) [4] diagnoses M00-M99, (MSDs) are still prevalent [5,6] and the second largest cause of productivity loss after mental ill-health [7]. The global burden estimates for MSD are high among working-age populations [8]. Thus, more knowledge is needed on the effect of MSDs on working life participation to target prevention and care, but also to support a sustainable working life.

Until recently, population-based cohort studies have been scarce for working life patterns over the life course [9–12], and mainly limited to evaluating the working life status after MSD [13–15]. Hence, an urgent need is to identify the informative clusters of sustainable working life over time among those with MSD [16,17]. Another recent development has been the so-called data-driven methods [18], such as sequence analysis [9,11,12], that provide opportunities to understand detailed clusters and transitions over time. Hence to shed further light on sustainable working life among individuals with MSD, studies with comprehensive and good-quality register data are needed.

MSD, SA/DP and sustainable working life are all known to carry a genetic component [19–23]. Hence, the associations between MSD and a sustainable working life are assumed to be affected by genetics and, possibly, by shared, mainly childhood, environment. The role of genetics and shared environment (i.e. familial factors) can be evaluated in a twins study, which can compare the associations between monozygotic (MZ) twins, who share 100% of genes, with dizygotic (DZ) twins, who share on average 50% of their genetic make-up. If familial factors were important, that would be seen either in the associations within DZ twin pairs, but not within MZ twin pairs, or only between twin individuals but not within MZ or DZ pairs. The interpretation would be that actions at the early stages of life, instead of prevention or care in adulthood, might be needed. Instead, if the associations would remain within both MZ and DZ twin pairs, that would point towards a direct link from MSD to sustainable working life [24]. Thus, a twins study would add to the earlier knowledge based on unrelated populations [10] and provide an understanding of targeting and timing interventions.

## Aims

The aims of this Swedish twins cohort study were: 1) to investigate sustainable working life patterns via identification of time-related sequences of SA, DP and unemployment, and also taking into account old-age pension and premature death four years before and five years after the first MSD diagnosis; 2) to account for familial confounding (genetics and shared environment) via comparison of MZ and same-sexed DZ twin pairs for the sequences; 3) to examine the associations between sequences and sociodemographic characteristics including zygosity, education, degree of urbanization, and marital status.

## Methods

Data from the Swedish Twin Project of Disability Pension and Sickness Absence (STODS) were used including twins identified in the Swedish Twin Registry (STR) who were born between 1925 and 1990 (*N*=119,907 individuals). Data from national registers were linked to all twins in STODS: a) the STR was used to identify the twin population and for background information (zygosity, sex and birthyear); b) the Longitudinal Integrated Database for Health Insurance and Labour Market Studies of Statistics Sweden [25] was used for sociodemographic information (educational level, degree of urbanization, marital status), days of unemployment and old-age pension; c) the Micro Data for Analyses of Social Insurance database of the Swedish Social Insurance Agency for information on net days with SA and DP; d) the Causes of Death Register of the Swedish Board of Health and Welfare for dates of death; and the first incident inpatient- and specialized outpatient care episode with main or side diagnosis code (ICD-10) M00-M99 (Diseases of musculoskeletal system, MSD) from the National Patient Register held by the National Board of Health and Welfare.

For the analyses, this study population was first limited to individuals with an incident M00-M99 diagnosis (in any diagnosis field, i.e. being the main or side diagnosis) in inpatient- and specialized outpatient care during cohort entry (diagnosis year) between 1998 and 2015 (*N* = 49,926 with MSD). Including the unaffected co-twins for those twins who did not have a co-twin in the sample resulted in a sample of 74,653 individuals. Then, age at cohort entry was limited to 29–59 years, and only individuals who were resident in Sweden at baseline and who did not emigrate from Sweden during the study period were included. Individuals who died during the study period were also included. The final sample was 28,474 individuals. Out of the final sample, 19,940 had MSD, and 8534 were their co-twins without MSD (*n* of pairs = 11,944 and 4568 were twins without a co-twin). The final study sample included all twins regardless of their zygosity, but when testing the role of zygosity the analysis was limited to MZ and same-sexed DZ twin pairs only. The number of complete discordant (i.e. one twin with MSD and the co-twin without) was 8423 pairs, out of whom 6238 were MZ and DZ same-sex pairs.

### Design

Twins were followed annually four years before (T-4, T-3, T-2 and T-1, respectively) the first MSD diagnosis (T0) and five years after (T+1, T+2, T+3, T+4 and T+5, respectively), in total for 10 years. Healthy co-twins (i.e. a co-twin without MSD) were followed over the same period as their diagnosed twin and T0 for unaffected co-twins was the same as for the affected twin. Twin pairs in which both had an MSD were followed over their respective period from the date of the diagnosis.

### Individual characteristics

Sociodemographic characteristics were categorized as sex (men, women), age across 10-year groups from 24 years of age; zygosity (MZ; DZ same-sex; DZ opposite-sex and unknown zygosity), educational level (elementary school (⩽9 years)/missing; high school (10–12 years); university/collage (>12 years)), degree of urbanization [26] (cities; towns and suburbs; rural areas) and marital status (married; not married). These characteristics were measured the year before follow-up started (before T-4).

### Statistical methods

To explore patterns of interruptions in working life four years before (T-4 to T-1) the first incident MSD (T0 ) and for the five years after T0 (T1–5), we conducted a sequence analysis [9]. The sequences consisted of 10 yearly observations in a seven-element state space. The states were defined based on SA, DP, unemployment, death and old-age pension, and named according to the definitions below:

1. Sustainable working life (SWL): SA/DP 0–29 days and unemployment 0–90 days.
2. Unemployment >90 days: SA/DP 0–30 days and unemployment > 90 days.
3. Moderate SA/DP: SA/DP 30–179 days.
4. Almost full year of SA/DP: SA/DP 180–365 days.
5. Full year of SA/DP: SA/DP ⩾ 365 days.
6. Death.
7. Old-age pension.

When assigning states to the individual yearly observations, we utilized a prioritizing in which death was given priority over old-age pension and old-age pension over SA, DP and unemployment. Consequently, an individual was assigned the state of death if he/she died during the year or was already dead at the beginning of the year. Next, the state of old-age pension was assigned if the individual had an old-age pension >50% of the annual income during the year. Thereafter the remaining states (SA, DP and unemployment) were assigned. The unemployment state captures individuals with a high amount of unemployment and simultaneously low amounts of SA/DP. However, a low level of unemployment (⩽90 days) may also be present in other states including SWL and states with >30 days of SA/DP.

The inter-sequence distances were calculated by the optimal matching algorithm (Needleman–Wunsch), using a substitution matrix created based on observed transition rates between states. When creating the substitution matrix, the old-age pension state was left out of the calculations. The substitution costs related to the old-age pension state were then set to 0. This makes old-age pension neutral to the other states. The idea of making old-age pension neutral to the other states was that old-age pension is an exit from working life, and we want to focus on interruptions in working life. The insertion or deletion (indel) cost was set to 1.

We then proceeded with an agglomerative hierarchical cluster analysis to find clusters of similar individual sequences of interruptions in working life. The cluster solutions were calculated by Ward’s linkage method, using the pairwise distance matrix from the sequence analysis. To determine the optimal number of clusters, a variety of quality measures were computed and compared for cluster solutions containing up to 10 clusters (Supplemental material Figure S1 and Table S1 online).

For the identified clusters, we applied conditional logistic regression modelling to analyse the associations between cluster membership and individual characteristics. Conditional regression applies to a discordant twin pair analysis as twins in a pair with the same value on the dependent variable (belonging to a cluster) are dropped because they do not affect the estimate. Thus, the model identifies the associations based on twin differences.

Then we estimated the effect of familial confounding (i.e. genetics, and shared environment mainly in childhood) on clusters by comparing whether MZ twin pairs more often than same-sex DZ twin pairs were found in the same cluster with the logistic regression.

Furthermore, we ran the zygosity stratified conditional logistic regression analyses for the factors of interest. If the estimates based on MZ and same-sexed DZ pairs differ from the estimates of all twin pairs genetic and shared environmental factors are indicated. If there would be a stronger association among DZ twin pairs than MZ twin pairs this would indicate genetic effects, whereas stronger associations among MZ twin pairs compared with DZ twin pairs would indicate the opposite, that is, towards individual environmental effects.

All the analyses were performed with R version (4.3.1) and utilizing the packages cluster, TraMineR, survival, and WeightedCluster.

### Research ethics and consent to participate

The study protocol was designed and performed according to the principles of the Helsinki Declaration. The ethical vetting was performed and approved by the Regional Ethical Review Board of Stockholm, Sweden (Dnr: 2007/524-31, 2010/1346-32/5, 2014/311-32, 2015/1809-32, 2017/128-32). For this type of study formal consent is not required as this is a register-based study involving secondary analyses of already existing data.

## Results

**Figure 1.**
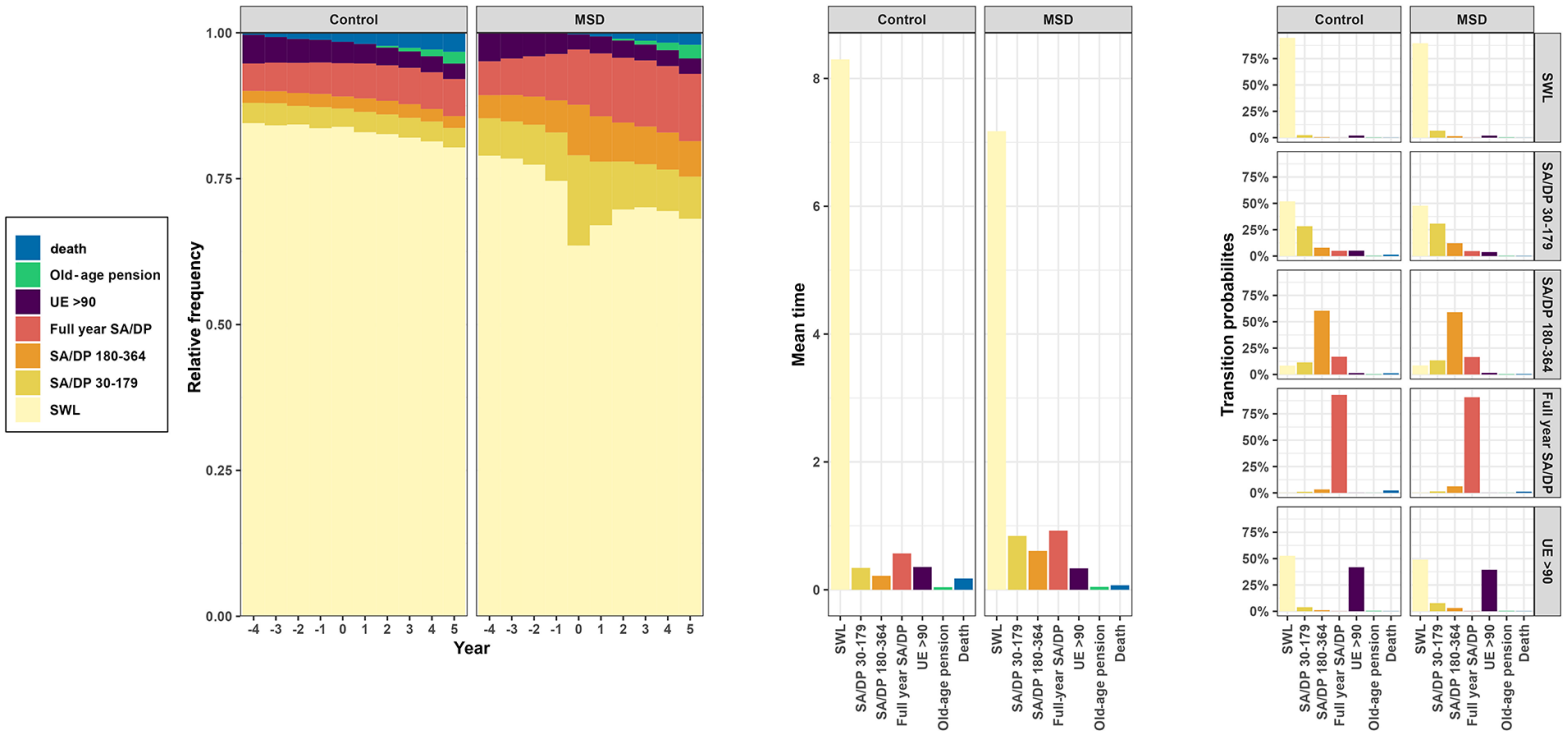
State distributions, mean state duration, and transition probabilities of those with musculoskeletal disorder (MSD) and their co-twins without MSD (control). The left-hand panel is the plot of state distributions, the middle panel is the mean time (in years) spent in each state, and the right-hand panel is transition probabilities between states. UE: unemployment; SA/DP: sickness absence/disability pension; SWL: sustainable working life

The final sample of 28,474 individuals included 19,940 twins with MSD and their respective co-twins in a twin pair (*n*=8 534, controls) (Figure 1). The state distributions for the pre-defined states before the T0 (time of MSD, i.e. four years) and after (five years) and mean time (in years) in each state (SWL, SA, DP, unemployment, old-age pension, or death) show differences between those with MSD and their co-twins without (controls). See Supplemental Table S2 for mean state durations. The transition probabilities between states are shown in Figure 1 in the right-hand panel and indicate that those with MSD and their co-twins (controls) have similar patterns (Supplemental Table S3 shows the percentage for probabilities). Transitioning from the moderate (30–179 days) of SA/DP to SWL is 50%, whereas transitioning from 180–364 days of SA/DP to SWL is 12% and from unemployment (>90 days) to SWL >40%. Those with full-year SA/DP have a probability of 95% remaining at that stage. The respective panels for the whole cohort are in the Supplemental material, Figure S2.

**Figure 2.**
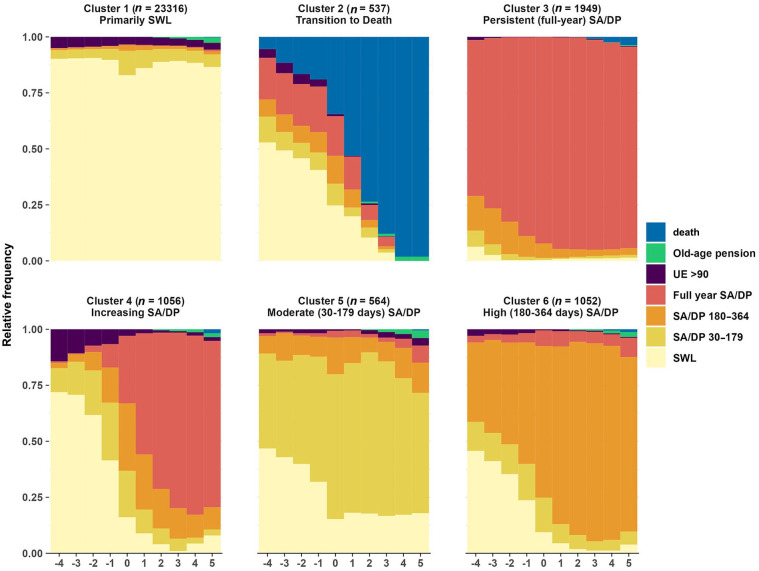
Plot of state distributions by cluster. UE: unemployment; SA/DP: sickness absence/disability pension; SWL: sustainable working life

The six-cluster solution had the best fit to the data (Supplemental Figure S1 and Table S1) and we identified five clusters with varying patterns of interruptions of sustainable working life (Clusters 2–6, *n* = 537–1949) compared with Cluster 1 with primarily SWL and being the largest (*n* = 23,316) (Figure 2). We then named the clusters Cluster 1: Primarily SWL, Cluster 2: Transition to death, Cluster 3: Persistent (full-year) SA/DP, Cluster 4: Increasing SA/DP, Cluster 5: Moderate (30–179 days) SA/DP, and Cluster 6: High (180–364 days) SA/DP. In Clusters 3 and 4, levels of SA/DP are high even before MSD, whereas in Clusters 2, 4 and 6, there is a high increase after MSD.

The plots for all sequences of the identified clusters are presented in Supplemental Figure S3.

Table I shows the descriptive characteristics of the clusters. All the clusters consisted mostly of individuals with MSD. However, the proportion of women, age groups and educational level differed across clusters, whereas the distributions of the degree of urbanization (for residential areas), marital status or zygosity were rather similar across identified clusters.

**Table I.** Descriptive characteristics (frequency and percentage) of the six identified clusters.

	Cluster 1^a^	Cluster 2^a^	Cluster 3^a^	Cluster 4^a^	Cluster 5^a^	Cluster 6^a^	All
	(*N*=23,316)	(*N*=537)	(*N*=1949)	(*N*=1056)	(*N*=564)	(*N*=1052)	(*N*=28,474)
MSD	*n* (%)	*n* (%)	*n* (%)	*n* (%)	*n* (%)	*n* (%)	*n* (%)
Control	7557 (32.4%)	229 (42.6%)	429 (22.0%)	128 (12.1%)	62 (11.0%)	129 (12.3%)	8534 (30.0%)
MSD	15759 (67.6%)	308 (57.4%)	1520 (78.0%)	928 (87.9%)	502 (89.0%)	923 (87.7%)	19,940 (70.0%)
**Sex**							
Men	12,051 (51.7%)	329 (61.3%)	695 (35.7%)	443 (42.0%)	160 (28.4%)	366 (34.8%)	14,044 (49.3%)
Women	11,265 (48.3%)	208 (38.7%)	1254 (64.3%)	613 (58.0%)	404 (71.6%)	686 (65.2%)	14,430 (50.7%)
**Age**							
24–34 years	6897 (29.6%)	57 (10.6%)	229 (11.7%)	215 (20.4%)	107 (19.0%)	130 (12.4%)	7635 (26.8%)
35–44 years	7848 (33.7%)	114 (21.2%)	516 (26.5%)	270 (25.6%)	143 (25.4%)	296 (28.1%)	9187 (32.3%)
45–54 years	8571 (36.8%)	366 (68.2%)	1204 (61.8%)	571 (54.1%)	314 (55.7%)	626 (59.5%)	11,652 (40.9%)
**Degree of urbanization**							
Cities	8602 (36.9%)	185 (34.5%)	658 (33.8%)	317 (30.0%)	188 (33.3%)	287 (27.3%)	10,237 (36.0%)
Towns and suburbs	9903 (42.5%)	222 (41.3%)	837 (42.9%)	446 (42.2%)	240 (42.6%)	476 (45.2%)	12,124 (42.6%)
Rural areas	4811 (20.6%)	130 (24.2%)	454 (23.3%)	293 (27.7%)	136 (24.1%)	289 (27.5%)	6113 (21.5%)
**Marital status**							
Other (single, separated or widowed)	12,947 (55.5%)	346 (64.4%)	1277 (65.5%)	597 (56.5%)	318 (56.4%)	531 (50.5%)	16,016 (56.2%)
Married/civil union	10,369 (44.5%)	191 (35.6%)	672 (34.5%)	459 (43.5%)	246 (43.6%)	521 (49.5%)	12,458 (43.8%)
**Educational level**							
0–9 years	3421 (14.7%)	171 (31.8%)	811 (41.6%)	334 (31.6%)	104 (18.4%)	279 (26.5%)	5120 (18.0%)
10–12 years	12,087 (51.8%)	273 (50.8%)	951 (48.8%)	588 (55.7%)	318 (56.4%)	595 (56.6%)	14,812 (52.0%)
>12 years	7808 (33.5%)	93 (17.3%)	187 (9.6%)	134 (12.7%)	142 (25.2%)	178 (16.9%)	8542 (30.0%)
**Zygosity**							
Monozygotic	5485 (23.5%)	101 (18.8%)	372 (19.1%)	227 (21.5%)	131 (23.2%)	227 (21.6%)	6543 (23.0%)
Dizygotic	6668 (28.6%)	159 (29.6%)	559 (28.7%)	287 (27.2%)	187 (33.2%)	342 (32.5%)	8202 (28.8%)
Opposite-sexed dizygotic and unknown zygosity	11,163 (47.9%)	277 (51.6%)	1018 (52.2%)	542 (51.3%)	246 (43.6%)	483 (45.9%)	13,729 (48.2%)

a Cluster 1: Primarily SWL, Cluster 2: Transition to death, Cluster 3: Persistent (full-year) SA/DP, Cluster 4: Increasing SA/DP, Cluster 5: Moderate (30–179 days) SA/DP, Cluster 6: High (180–364 days) SA/DP Statistically significant OR with 95%CI in boldface.

MSD: musculoskeletal disorder; SA/DP: sickness absence/disability pension; SWL: sustainable working life

The assessment of the associations of sociodemographic factors on the likelihood of cluster memberships among the whole cohort (Table II) indicated that in comparison with Cluster 1 (SWL), having MSD or being a woman increased the odds of belonging to Clusters 3–6. Older age was associated with higher odds of belonging to Cluster 3 (Persistent (full-year) SA/DP), whereas the likelihood of belonging to Clusters 4 (Increasing SA/DP) and 6 (High SA/DP) was lower. Being married decreased the odds of belonging to Clusters 2–4, and higher education to Clusters 2–3. The respective analysis using any other cluster as a reference is in Supplemental Table S4. A comparison of associations between MZ and same-sex DZ twins (Table III) for sociodemographic factors and the likelihood of cluster memberships indicated that most associations retained the direction and magnitude. Thus, we cannot rule out familial confounding.

**Table II.** Conditional logistic regression for odds ratios (ORs) and 95% confidence intervals (CIs) for associations between sociodemographic factors and cluster memberships while having Cluster 1 as reference (ref.).

	Comparison with Cluster 1^a^									
Characteristic	Cluster 2^a^		Cluster 3^a^		Cluster 4^a^		Cluster 5^a^		Cluster 6^a^	
	OR	95% CI	OR	95% CI	OR	95% CI	OR	95% CI	OR	95% CI
**MSD**										
Control	ref.	—	ref.	—	ref.	—	ref.	—	ref.	—
MSD	0.77	0.59–1.01	**1.81**	**1.50–2.18**	**3.86**	**2.94–5.07**	**3.56**	**2.48–5.11**	**3.22**	**2.50–4.15**
**Sex**										
Men	ref.	—	ref.	—	ref.	—	ref.	—	ref.	—
Women	0.81	0.56–1.19	**2.26**	**1.76–2.89**	**1.59**	**1.14–2.21**	**3.53**	**2.22–5.61**	**2.38**	**1.71–3.32**
**Age**										
24–34 years	ref.	—	ref.	—	ref.	—	ref.	—	ref.	—
35–44 years	0.62	0.03–11.6	**2.86**	**1.31–6.25**	**0.17**	**0.06–0.51**	0.81	0.26–2.48	0.62	0.27–1.45
45–54 years	0.88	0.04–18.6	**2.81**	**1.11–7.09**	**0.06**	**0.02–0.21**	0.61	0.16–2.31	**0.33**	**0.12–0.88**
**Residential regions**										
Cities	ref.	—	ref.	—	ref.	—	ref.	—	ref.	—
Towns and suburbs	0.93	0.53–1.63	0.75	0.54–1.04	1.00	0.62–1.62	**0.48**	**0.28–0.83**	0.90	0.59–1.37
Rural areas	0.76	0.40–1.42	1.01	0.68–1.52	1.17	0.67–2.05	0.62	0.32–1.21	1.14	0.69–1.91
**Marital status**										
Other	ref.	—	ref.	—	ref.	—	ref.	—	ref.	—
Married/civil union	**0.61**	**0.43–0.87**	**0.38**	**0.30–0.49**	**0.68**	**0.49–0.94**	0.69	0.46–1.02	0.90	0.68–1.20
**Education**										
0–9 years	ref.	—	ref.	—	ref.	—	ref.	—	ref.	—
10–12 years	0.65	0.40–1.04	**0.47**	**0.36–0.62**	0.98	0.65–1.49	0.91	0.52–1.58	1.06	0.73–1.56
>12 years	**0.41**	**0.21–0.79**	**0.13**	**0.08–0.20**	0.73	0.40–1.32	0.81	0.41–1.63	0.74	0.43–1.26

a Cluster 1: Primarily SWL, Cluster 2: Transition to death, Cluster 3: Persistent (full-year) SA/DP, Cluster 4: Increasing SA/DP, Cluster 5: Moderate (30–179 days) SA/DP, Cluster 6: High (180–364 days) SA/DP. Statistically significant OR with 95%CI in boldface.

MSD: musculoskeletal disorder; SA/DP: sickness absence/disability pension; SWL: sustainable working life

**Table III.** Conditional logistic regression across monozygotic (MZ) and same-sexed dizygotic (DZ) twins for odds ratios (ORs) and 95% confidence intervals (CIs) for associations between sociodemographic factors and cluster memberships while having any other cluster as reference (ref.).

MZ	Cluster 1^a^			Cluster 2^a^			Cluster 3^a^			Cluster 4a			Cluster 5^a^			Cluster 6^a^		
	Event rate	OR	95% CI	Event rate	OR	95% CI	Event rate	OR	95% CI	Event rate	OR	95% CI	Event rate	OR	95% CI	Event rate	OR	95% CI
**MSD**																		
Control	1635/1808 (90%)	ref.	—	42/1808 (2.3%)	ref.	—	82/1808 (4.5%)	ref.	—	18/1808 (1.0%)	ref.	—	8/1808 (0.44%)	ref.	—	23/1808 (1.3%)	ref.	—
MSD	3850/4735 (81%)	**0.47**	**0.36–0.61**	59/4735 (1.2%)	**0.45**	**0.24–0.85**	290/4735 (6.1%)	0.83	0.57–1.23	209/4735 (4.4%)	**3.87**	**2.29–6.54**	123/4735 (2.6%)	**3.96**	**1.79–8.75**	204/4735 (4.3%)	**3.39**	**2.03–5.65**
**Residential regions**																		
Cities	2101/2419 (87%)	ref.	—	33/2419 (1.4%)	ref.	—	115/2419 (4.8%)	ref.	—	65/2419 (2.7%)	ref.	—	41/2419 (1.7%)	ref.	—	64/2419 (2.6%)	ref.	—
Towns and suburbs	2345/2822 (83%)	1.23	0.77–1.98	47/2822 (1.7%)	1.82	0.50–6.59	167/2822 (5.9%)	0.93	0.43–2.01	90/2822 (3.2%)	0.79	0.33–1.89	59/2822 (2.1%)	0.59	0.23–1.54	114/2822 (4.0%)	0.86	0.36–2.03
Rural areas	1039/1302 (80%)	0.78	0.46–1.33	21/1302 (1.6%)	0.88	0.17–4.46	90/1302 (6.9%)	1.20	0.50–2.87	72/1302 (5.5%)	1.37	0.54–3.45	31/1302 (2.4%)	0.63	0.18–2.17	49/1302 (3.8%)	0.96	0.34–2.73
**Marital status**																		
Other	3037/3610 (84%)	ref.	—	61/3610 (1.7%)	ref.	—	224/3610 (6.2%)	ref.	—	115/3610 (3.2%)	ref.	—	63/3610 (1.7%)	ref.	—	110/3610 (3.0%)	ref.	—
Married/civil union	2448/2933 (83%)	1.19	0.87–1.62	40/2933 (1.4%)	**0.34**	**0.14–0.84**	148/2933 (5.0%)	0.81	0.50–1.32	112/2933 (3.8%)	1.06	0.60–1.88	68/2933 (2.3%)	0.90	0.45–1.81	117/2933 (4.0%)	1.25	0.72–2.15
**Education**																		
0–9 years	632/897 (70%)	ref.	—	15/897 (1.7%)	ref.	—	130/897 (14%)	ref.	—	65/897 (7.2%)	ref.	—	15/897 (1.7%)	ref.	—	40/897 (4.5%)	ref.	—
10–12 years	2765/3374 (82%)	0.83	0.52–1.34	61/3374 (1.8%)	1.07	0.26–4.42	193/3374 (5.7%)	0.67	0.36–1.27	133/3374 (3.9%)	0.93	0.41–2.12	81/3374 (2.4%)	1.34	0.42–4.29	141/3374 (4.2%)	2.00	0.84–4.77
>12 years	2088/2272 (92%)	**2.16**	**1.10–4.22**	25/2272 (1.1%)	**0.06**	**0.00–0.80**	49/2272 (2.2%)	**0.16**	**0.05–0.51**	29/2272 (1.3%)	0.53	0.13–2.11	35/2272 (1.5%)	1.87	0.44–7.97	46/2272 (2.0%)	1.42	0.44–4.66
**DZ**	**Event rate**	**OR**	**95% CI**	**Event rate**	**OR**	**95% CI**	**Event rate**	**OR**	**95% CI**	**Event rate**	**OR**	**95% CI**	**Event rate**	**OR**	**95% CI**	**Event rate**	**OR**	**95% CI**
**MSD**																		
Control	2267/2565 (88%)	ref.	—	64/2565 (2.5%)	ref.	—	113/2565 (4.4%)	ref.	—	40/2565 (1.6%)	ref.	—	31/2565 (1.2%)	ref.	—	50/2565 (1.9%)	ref.	—
MSD	4401/5637 (78%)	**0.48**	**0.40–0.57**	95/5637 (1.7%)	0.78	0.50–1.20	446/5637 (7.9%)	**1.86**	**1.39–2.49**	247/5637 (4.4%)	**2.37**	**1.59–3.53**	156/5637 (2.8%)	**2.17**	**1.39–3.40**	292/5637 (5.2%)	**2.55**	**1.78–3.65**
**Residential regions**																		
Cities	2412/2922 (83%)	ref.	—	56/2922 (1.9%)	**ref.**	—	200/2922 (6.8%)	ref.	—	92/2922 (3.1%)	ref.	—	65/2922 (2.2%)	ref.	—	97/2922 (3.3%)	ref.	—
Towns and suburbs	2829/3459 (82%)	1.29	0.94–1.78	68/3459 (2.0%)	1.84	0.76–4.45	220/3459 (6.4%)	**0.56**	**0.35–0.90**	121/3459 (3.5%)	1.25	0.61–2.57	74/3459 (2.1%)	**0.39**	**0.17–0.88**	147/3459 (4.2%)	0.99	0.55–1.78
Rural areas	1427/1821 (78%)	1.29	0.89–1.88	35/1821 (1.9%)	1.02	0.41–2.50	139/1821 (7.6%)	0.66	0.38–1.17	74/1821 (4.1%)	1.19	0.57–2.49	48/1821 (2.6%)	0.56	0.22–1.42	98/1821 (5.4%)	1.35	0.69–2.67
**Marital status**																		
Other	3392/4230 (80%)	ref.	—	105/4230 (2.5%)	ref.	—	337/4230 (8.0%)	ref.	—	139/4230 (3.3%)	ref.	—	95/4230 (2.2%)	ref.	—	162/4230 (3.8%)	ref.	—
Married/civil union	3276/3972 (82%)	**1.74**	**1.39–2.17**	54/3972 (1.4%)	**0.34**	**0.18–0.62**	222/3972 (5.6%)	**0.56**	**0.40–0.79**	148/3972 (3.7%)	0.81	0.52–1.26	92/3972 (2.3%)	1.00	0.59–1.69	180/3972 (4.5%)	1.05	0.70–1.58
**Education**																		
0–9 years	1012/1517 (67%)	ref.	—	51/1517 (3.4%)	ref.	—	231/1517 (15%)	ref.	—	94/1517 (6.2%)	ref.	—	33/1517 (2.2%)	ref.	—	96/1517 (6.3%)	ref.	—
10–12 years	3377/4172 (81%)	**1.54**	**1.15–2.06**	81/4172 (1.9%)	0.63	0.30–1.30	269/4172 (6.4%)	**0.61**	**0.41–0.90**	152/4172 (3.6%)	0.69	0.37–1.27	101/4172 (2.4%)	1.67	0.78–3.59	192/4172 (4.6%)	1.15	0.70–1.89
>12 years	2279/2513 (91%)	**3.10**	**2.07–4.63**	27/2513 (1.1%)	0.66	0.25–1.76	59/2513 (2.3%)	**0.21**	**0.11–0.39**	41/2513 (1.6%)	0.69	0.30–1.59	53/2513 (2.1%)	1.56	0.60–4.07	54/2513 (2.1%)	0.52	0.24–1.12

a Cluster 1: Primarily SWL, Cluster 2: Transition to death, Cluster 3: Persistent (full-year) SA/DP, Cluster 4: Increasing SA/DP, Cluster 5: Moderate (30–179 days) SA/DP, Cluster 6: High (180–364 days) SA/DP Statistically significant OR with 95%CI in boldface.

MSD: musculoskeletal disorder; SA/DP: sickness absence/disability pension; SWL: sustainable working life

The analysis for the likelihood of both twins belonging to the same cluster across zygosity (MZ or same-sexed DZ) indicated that, overall, DZ had odds ratio (OR) 0.72 (95% confidence interval (CI) 0.64, 0.81) compared with MZ, which was indicative of a lack of familial factors. The association retained magnitude and direction in the cluster-specific analyses for Cluster 1 but could not be confirmed in other clusters likely affected by power issues (Supplemental Table S5). The comparison of concordant (both twins had MSD) versus discordant (only one of the twins had MSD) twin pairs indicated concordant DZ to have OR 0.72 (95% CI 0.60, 0.88), discordant MZ 1.74 (1.45, 2.10) and discordant DZ 1.17 (0.99, 1.39) in comparison with concordant MZ pairs, also pointing towards independency from familial factors.

## Discussion

In this prospective study of 28,474 Swedish twins, we aimed to investigate SWL patterns via the identification of time-related sequences of SA, DP and unemployment, while accounting for death and old-age pension four years before and five years after the first MSD diagnosis (*n* = 19,940 twins including also twins of whom both within a twin pair had MSD) whereas the twin sibling without MSD was followed for comparison (*n*=8534). The largest cluster had 23,316 twins who had primarily SWL, whereas the other identified five clusters had varying sizes (*n* = 537–1949) and patterns of interruptions of SWL. The transition probabilities were similar for those with and without MSD and indicated that the transition to SWL was highest from moderate (30–179 days) SA/DP (48–52%) and unemployment (49–53%), whereas it was around 8.5% from almost full-year (180–364 days) SA/DP. Furthermore, the use of twins provided us with the opportunity to account for familial confounding, which seemed to influence the cluster memberships. In addition, the associations between sequences and sociodemographic characteristics, including zygosity, education, degree of urbanization and marital, showed that in comparison with Cluster 1, having MSD or being a woman increased the odds of belonging to Clusters 3–6, that is, those with persistent, increasing, moderate or high SA/DP. Older (⩾35 years) age was associated with higher odds of belonging to Cluster 3 (Persistent (full year) SA/DP), whereas the likelihood of belonging to Clusters 4 and 6 was lower. Also, marital status decreased the odds of belonging to Cluster 2: Transition to death, Cluster 3: Persistent (full-year) SA/DP, and Cluster 4: Increasing SA/DP, while higher education decreased the odds for Clusters 2–3. This adds to the earlier, but scarce, knowledge based on population-based cohort studies [9–11] and among those with MSD [16,17].

We detected Clusters 3 and 4 with high levels of SA/DP already before MSD, which implies that having either recurrent or long SA/DP could be considered a ‘red flag’ calling for health assessment and preventative actions. This is consistent with the previous knowledge related to the burden of MSD, which is known to be high among working-age populations [8]. However, prevalent MSD may also imply that working with such chronic conditions can be beneficial and/or symptoms affecting participation in paid work might be in control. Consequently, this suggests that prevention and interventions targeting MSD (such as prescribed physical activity) should be provided early, and could be provided at workplaces and (occupational) health care, and highlighted at policy-level decisions.

Being a woman or ⩾35 years of age was associated with a higher likelihood of belonging to the clusters with persistent, increasing, moderate and high SA/DP, whereas MSD itself was the strongest predictor. First, the marked role of MSD emphasizes the need to prevent and treat MSD early. Second, our findings call for targeted interventions and actions that could address the specific needs of women or middle-aged employees. Further studies should elaborate on whether such actions could be occupational sector- or occupational group-specific [27] as they might play an additional role not assessed in this study. Our results point towards the importance of environmental factors (being independent from familial factors), as the results for MZ and DZ twins remained and they seemed to play a role in the cluster memberships. Although this finding should be ascertained in future studies, it is known that there might be specific genetic susceptibility or the known heritability of other influential factors such as body mass index [28] and pain [29] affecting the associations between MSD and SWL. Our finding aligns with some earlier findings based on the Nordic twin cohorts with SA/DP data [21,23]. The role of genetics emphasizes the early prevention and treatment of MSD but also implies that tailored interventions might be needed, that is, for women, older employees, and occupational groups.

This study had several strengths as we had access to comprehensive, good-quality national register data without loss to follow-up or reporting bias. Furthermore, the use of twins enabled the assessment of genetic similarity, which added to the previous research on MSD and SWL. Yet, the detailed register data provided the possibility to apply data-driven methodology, and sequence analysis to assess various states and transitions between them across 10 years of follow-up. Such a long follow-up has relatively rarely been used before [9,11]. Although before–after assessment of working life trajectories have been done before, for example, related to surgery or infarction [30,31], we are not aware of a similar approach as in this study to assess states and transitions four years before MSD and five years after in a population-based sample of twins. All studies also have weaknesses. In this study, MSD was highly prevalent in all clusters. This might be due to the construction of the study sample. For example, the number of twins may be unmatched due to data restrictions, which would inflate the relative number of MSD cases. Twin pairs where both have MSD did not have healthy controls in this study, hence there were more MSD cases than healthy controls overall and by cluster. Hence, even a larger sample might be warranted to test these assumptions. Furthermore, as we included all MSD diagnoses known to be heterogeneous [32,33], severity and persistence in terms of functional impairment were not accounted for in our study. That may have diluted the results and is why further studies should investigate the MSD diagnosis groups in more detail. Second, we utilized only register data, which may have limited the evaluation of potentially relevant, influential factors such as occupational social class, psychosocial working conditions, health behaviours or other factors [34,35]. These factors, some of them only accessible via survey data, should be addressed in future studies. Another weakness might be the context since we utilized Swedish register data. This might limit the generalization, less to other Nordic countries that have similar society and welfare state models, but more to other countries.

## Conclusions

Most Swedish twins with or without MSD have a SWL; that is, no, or very few, interruptions due to SA/DP or unemployment during working life. However, MSD was both prevalent and a strong risk factor for belonging to the clusters with SA/DP, although we also identified clusters with high SA/DP even before being diagnosed with MSD. All in all, early prevention of MSD and prevention of recurrent or long sickness absences due to any cause would be merited. Women should also be addressed with targeted actions to maintain a SWL.

## Supplemental Material

sj-docx-1-sjp-10.1177_14034948241284041 – Supplemental material for Sequences of sickness absence, disability pension and unemployment four years before and five years after musculoskeletal diagnosis among Swedish twins Supplemental material, sj-docx-1-sjp-10.1177_14034948241284041 for Sequences of sickness absence, disability pension and unemployment four years before and five years after musculoskeletal diagnosis among Swedish twins by Annina Ropponen, Emma Pettersson, Jurgita Narusyte and Pia Svedberg in Scandinavian Journal of Public Health
