# Supplementary material for: Sequences of sickness absence, disability pension and unemployment four years before and five years after musculoskeletal diagnosis among Swedish twins
Source: Scand J Public Health. 2024 Oct 31;54(1):24–33. doi: 10.1177/14034948241284041 (PMC12858660; doi:10.1177/14034948241284041)
Supplement: sj-docx-1-sjp-10.1177_14034948241284041 – Supplemental material for Sequences of sickness absence, disability pension and unemployment four years before and five years after musculoskeletal diagnosis among Swedish twins [file sj-docx-1-sjp-10.1177_14034948241284041.docx]

**Supplemental material**


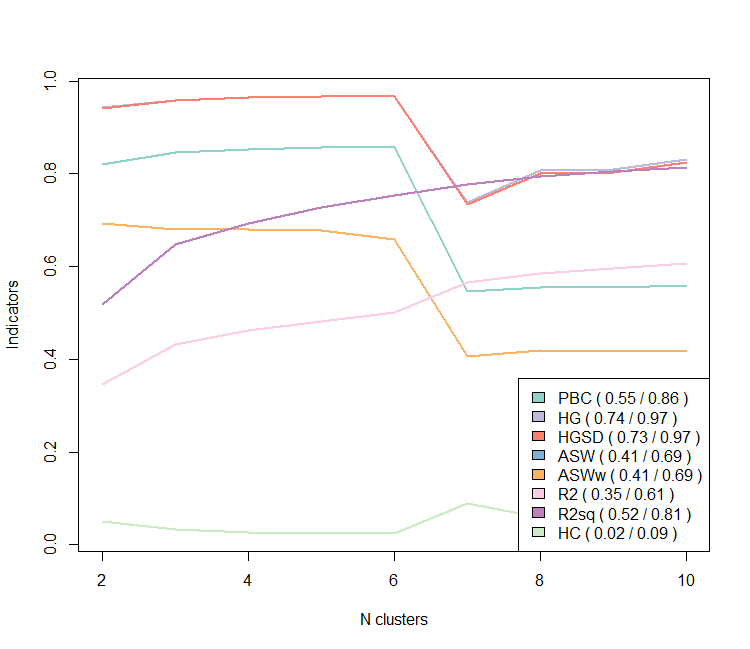


**Supplemental Figure S1.** Measures of goodness of fit for the best number of clusters

**Supplemental Table S1** Optimal cluster solution based on different quality measures

|  |  | N groups | statistics |
| --- | --- | --- | --- |
| **Point Biserial Correlation** | **PBC** | **6** | **0.8580** |
| **Hubert's Gamma** | **HG** | **6** | **0.9676** |
| **Hubert's Somers D** | **HGSD** | **6** | **0.9673** |
| Average Silhouette Width | ASW | 2 | 0.6934 |
| Average Silhouette Width weighted | ASWw | 2 | 0.6935 |
| Calinski-Harabasz index | CH | 2 | 15026.9770 |
| Pseudo R2 | R2 | 10 | 0.6061 |
| Calinski-Harabasz index using squared distances | CHsq | 2 | 30707.3283 |
| Pseudo R2 using squared distances | R2sq | 10 | 0.8143 |
| **Hubert's C** | **HC** | **6** | **0.0243** |

The six cluster solution in boldface

**Supplemental Table S2** Mean State Duration of those with MSD and their co-twins without MSD (control).

| **State** | **MSD** | **Control** |
| --- | --- | --- |
|  | **Mean (years)** | **Mean**  **(years)** |
| SWL | 7.17 | 8.30 |
| SA/DP 30-179 | 0.84 | 0.34 |
| SA/DP 180-364 | 0.61 | 0.22 |
| Full year SA/DP | 0.93 | 0.57 |
| UE > 90 | 0.34 | 0.36 |
| Old age pension | 0.05 | 0.04 |
| Death | 0.07 | 0.18 |

**Supplemental Table S3** Transition Probabilities of those with MSD and their co-twins without MSD (control).

| **MSD** | | **To** | | | | | | |
| --- | --- | --- | --- | --- | --- | --- | --- | --- |
|  |  | **SWL** | **SA/DP**  **30-179** | **SA/DP**  **180-364** | **Full year**  **SA/DP** | **UE >90** | **Old**  **age pension** | **Death** |
| **From** | **SWL** | 89.6% | 6.7% | 1.4% | 0.1% | 2.0% | 0.3% | 0.1% |
|  | **SA/DP 30-179** | 47.8% | 30.8% | 12.2% | 4.7% | 3.8% | 0.3% | 0.3% |
|  | **SA/DP 180-364** | 8.6% | 13.4% | 59.1% | 16.6% | 1.5% | 0.3% | 0.5% |
|  | **Full year SA/DP** | 0.3% | 1.4% | 6.3 | 90.7% | 0.1% | 0.1% | 1.2% |
|  | **UE >90** | 49.1% | 7.7% | 3.1% | 0.2% | 39.4% | 0.3% | 0.2% |
| **Control** | |  | | | | | | |
| **From** | **SWL** | 94.8% | 2.4% | 0.5% | 0% | 2.0% | 0.2% | 0.1% |
|  | **SA/DP 30-179** | 52.0% | 28.3% | 7.9% | 5.0% | 5.1% | 0.3% | 1.3% |
|  | **SA/DP 180-364** | 8.4% | 11.5% | 60.5% | 16.9% | 1.3% | 0.3% | 1.1% |
|  | **Full year SA/DP** | 0.2% | 1.0% | 3.3% | 93.0% | 0.1% | 0.1% | 2.3% |
|  | **UE >90** | 52.7% | 3.9% | 1.1% | 0.1% | 41.8% | 0.4% | 0.1% |


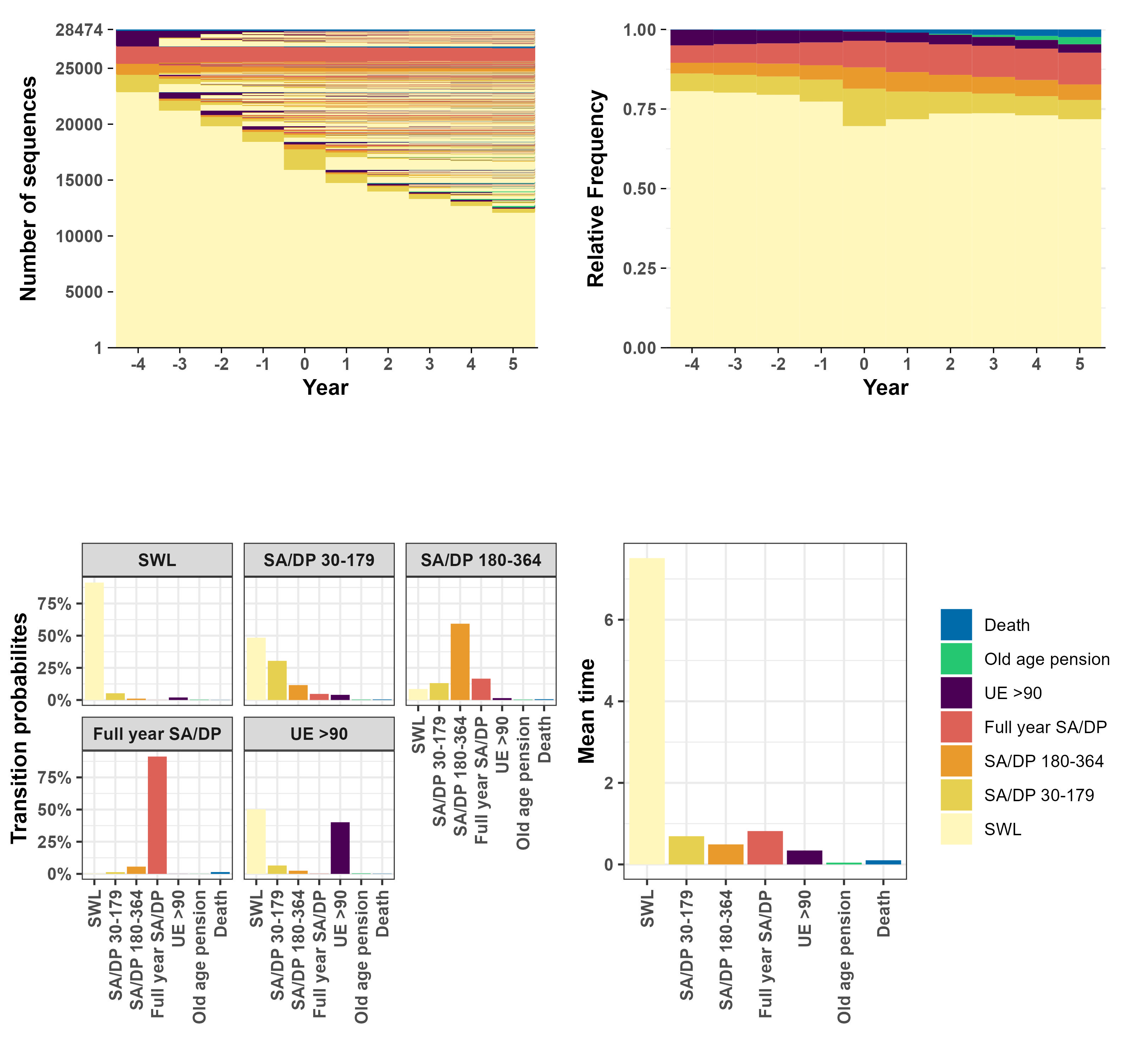


**
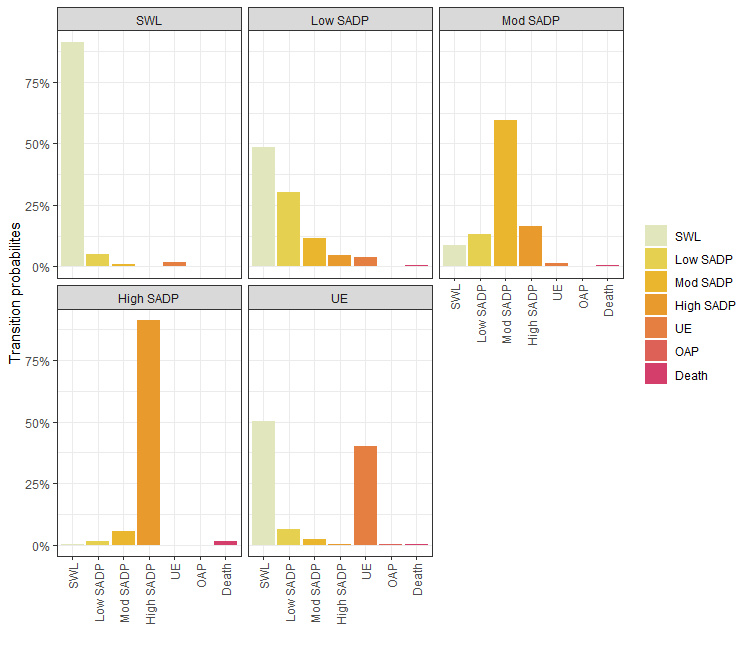
Supplemental figure S2.** The sequences of the whole cohort. On upper left-hand corner is the plot of all sequences, upper right-hand corner is plot of state distributions, lower right-hand corner is mean time (in years) spend in each cluster, and in the lower left-hand corner transition probabilities.


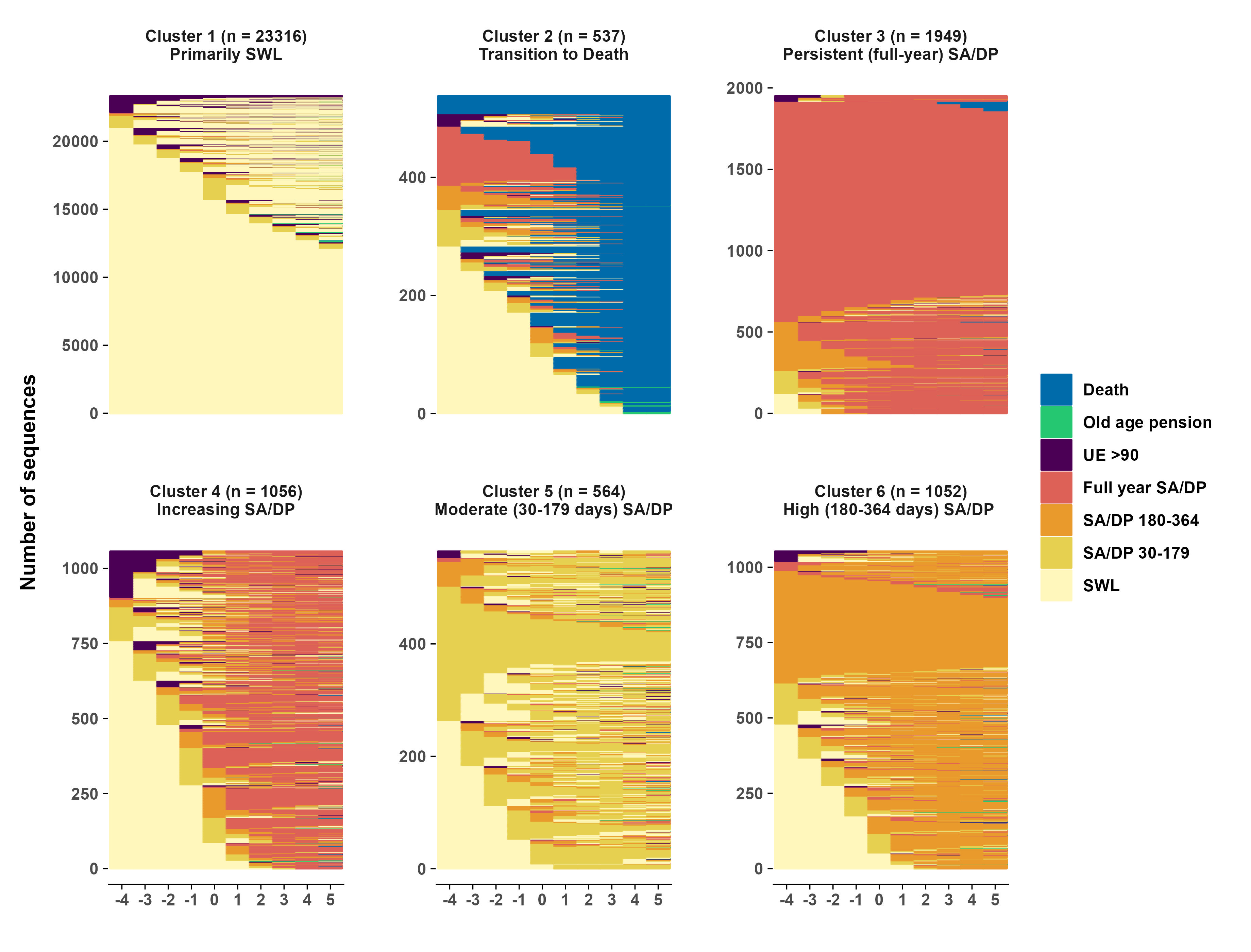


**Supplemental Figure S3.** Plot of all sequences by cluster

|  | **Cluster1** | | **Cluster2** | | **Cluster3** | | **Cluster4** | | **Cluster5** | | **Cluster6** | |
| --- | --- | --- | --- | --- | --- | --- | --- | --- | --- | --- | --- | --- |
|  | **OR** | **95% CI** | **OR** | **95% CI** | **OR** | **95% CI** | **OR** | **95% CI** | **OR** | **95% CI** | **OR** | **95% CI** |
| **MSD** |  |  |  |  |  |  |  |  |  |  |  |  |
| Control | ref | — | ref | — | ref | — | ref | — | ref | — | ref | — |
| MSD | **0.48** | **0.44-0.54** | **0.62** | **0.50-0.79** | **1.43** | **1.22-1.67** | **3.03** | **2.43-3.77** | **3.08** | **2.27-4.19** | **2.85** | **2.30-3.53** |
| **Sex** |  |  |  |  |  |  |  |  |  |  |  |  |
| Men | ref | — | ref | — | ref | — | ref | — | ref | — | ref | — |
| Women | **0.53** | **0.46-0.61** | **0.69** | **0.49-0.97** | **2.10** | **1.70-2.59** | 1.19 | 0.91-1.55 | **3.00** | **2.01-4.48** | **1.84** | **1.39-2.42** |
| **Age** |  |  |  |  |  |  |  |  |  |  |  |  |
| 24-34 years | ref | — | ref | — | ref | — | ref | — | ref | — | ref | — |
| 35-44 years | 1.20 | 0.79-1.80 | 0.99 | 0.12-8.00 | **1.90** | **1.00-3.61** | **0.50** | **0.25-1.00** | 0.83 | 0.32-2.19 | 0.50 | 0.24-1.05 |
| 45-54 years | **1.68** | **1.03-2.74** | 1.19 | 0.14-10.2 | 2.11 | 0.98-4.53 | **0.27** | **0.12-0.65** | 0.72 | 0.23-2.28 | **0.29** | **0.12-0.67** |
| **Residential regions** |  |  |  |  |  |  |  |  |  |  |  |  |
| Cities | ref | — | ref | — | ref | — | ref | — | ref | — | ref | — |
| Towns and suburbs | 1.16 | 0.96-1.39 | 1.00 | 0.62-1.62 | 0.76 | 0.58-1.00 | 0.99 | 0.68-1.43 | **0.52** | **0.32-0.83** | 1.08 | 0.76-1.55 |
| Rural areas | 1.03 | 0.82-1.28 | 0.81 | 0.46-1.41 | 0.87 | 0.63-1.22 | 1.24 | 0.80-1.92 | 0.70 | 0.40-1.24 | 1.23 | 0.80-1.89 |
| **Marital status** |  |  |  |  |  |  |  |  |  |  |  |  |
| Other (single, separated, widowed) | ref | — | ref | — | ref | — | ref | — | ref | — | ref | — |
| Married/civil union | **1.70** | **1.49-1.93** | **0.57** | **0.42-0.78** | **0.48** | **0.39-0.59** | 0.79 | 0.61-1.02 | 0.81 | 0.58-1.14 | 1.09 | 0.86-1.39 |
| **Education** |  |  |  |  |  |  |  |  |  |  |  |  |
| 0-9 years | ref | — | ref | — | ref | — | ref | — | ref | — | ref | — |
| 10-12 years | **1.50** | **1.27-1.76** | 0.73 | 0.49-1.09 | **0.55** | **0.44-0.68** | 0.98 | 0.72-1.33 | 1.15 | 0.72-1.83 | 1.14 | 0.84-1.55 |
| >12 years | **2.83** | **2.25-3.56** | **0.44** | **0.25-0.78** | **0.21** | **0.14-0.29** | 0.73 | 0.45-1.18 | 1.02 | 0.56-1.87 | 0.92 | 0.58-1.45 |

**Supplemental Table S4.** Conditional logistic regression - adjusted OR - cluster x vs any other clust**er**

**Supplemental Table S5** Logistic regression (odds ratio, OR and 95% confidence interval, CI) for the likelihood of both twins belonging to the same cluster across zygosity (MZ = monozygotic, DZ = dizygotic)

| **Characteristic** | **cluster1** | | | **cluster2** | | | **cluster3** | | | | **cluster4** | | | **cluster5** | | | **cluster6** | | |
| --- | --- | --- | --- | --- | --- | --- | --- | --- | --- | --- | --- | --- | --- | --- | --- | --- | --- | --- | --- |
|  | **Event Rate** | **OR** | **95% CI** | **Event Rate** | **OR** | **95% CI** | | **Event Rate** | **OR** | **95% CI** | **Event Rate** | **OR** | **95% CI** | **Event Rate** | **OR** | **95% CI** | **Event Rate** | **OR** | **95% CI** |
|  |  |  |  |  |  |  | |  |  |  |  |  |  |  |  |  |  |  |  |
| MZ - Both MSD | 653/952 (69%) | ref | — | 1/952 (0.11%) | ref | — | | 21/952 (2.2%) | ref | — | 4/952 (0.42%) | ref | — | 1/952 (0.11%) | ref | — | 4/952 (0.42%) | ref | — |
| DZ - Both MSD | 603/960 (63%) | **0.77** | **0.64-0.93** | 0/960 (0.00%) | na |  | | 11/960 (1.1%) | 0.51 | 0.24-1.05 | 5/960 (0.52%) | 1.24 | 0.33-5.03 | 1/960 (0.10%) | 0.99 | 0.04-25.1 | 3/960 (0.31%) | 0.74 | 0.15-3.38 |
| MZ - One MSD | 1426/1780 (80%) | **1.84** | **1.54-2.21** | 4/1780 (0.22%) | 2.14 | 0.32-41.9 | | 20/1780 (1.1%) | **0.50** | **0.27-0.94** | 0/1780 (0.00%) | na |  | 0/1780 (0.00%) | na |  | 3/1780 (0.17%) | 0.40 | 0.08-1.82 |
| DZ - One MSD | 1873/2546 (74%) | **1.27** | **1.08-1.50** | 1/2546 (0.04%) | 0.37 | 0.01- 9.46 | | 25/2546 (0.98%) | **0.44** | **0.25-0.80** | 5/2546 (0.20%) | 0.47 | 0.12-1.89 | 0/2546 (0.00%) | na |  | 5/2546 (0.20%) | 0.47 | 0.12-1.89 |

na = not assessable due to lack of observations
